# The association between insemination eligibility and reproductive performance of nulliparous heifers on subsequent body weight and milk production of primiparous Holstein cows

**DOI:** 10.3168/jdsc.2023-0372

**Published:** 2023-07-13

**Authors:** M.R. Lauber, P.M. Fricke

**Affiliations:** Department of Animal and Dairy Sciences, University of Wisconsin–Madison, Madison, WI 53706

## Abstract

The objective of this retrospective cohort study was to determine the association between insemination eligibility and reproductive performance of nulliparous heifers with subsequent body weight (BW) at 30 d in milk (DIM) and milk production at wk 4, 8, and 12 of lactation of primiparous cows. The final data set included 1,849 primiparous Holstein cows from a commercial dairy herd. Data extracted from a commercial dairy herd management software program included parent average predicted transmitting abilities (PTA), bovine respiratory disease (BRD) incidence, pregnancies per artificial insemination (P/AI) at first insemination as nulliparous heifers, BW at 30 DIM, percent mature body weight (%MBW), and mean weekly milk production at wk 4, 8, and 12 of lactation. Heifers were eligible for first insemination at 380 d of age and were detected in estrus and artificially inseminated with sexed semen. Quartiles based on BW at 30 DIM and %MBW were created in ascending order as follows: Q1 (lightest; n = 462), Q2 (light-moderate; n = 456), Q3 (moderate; n = 472), and Q4 (heaviest; n = 459). Only Q3 and Q4 cows achieved ≥85% MBW postcalving based on the herd MBW of 686.2 kg. The incidence of BRD during heifer development was greatest for Q1 cows, and Q1 cows had fewer days on feed as heifers than Q4 cows, resulting in a shorter growth period to achieve ≥85% MBW postcalving. Overall, Q1 cows had a greater PTA for daughter pregnancy rate and heifer conception rate than Q4 cows, which was associated with approximately 26 percentage points more P/AI at first insemination as heifers for Q1 than for Q4 cows. Finally, Q1 cows yielded approximately 5 kg per cow/d less milk than Q4 cows. Thus, insemination eligibility and reproductive performance of heifers, calfhood BRD incidence, and genetic potential for reproductive performance were associated with BW at 30 DIM and milk production at wk 4, 8, and 12 of lactation of primiparous cows.

## Highlights

• Increased milk production was associated with heavier primiparous cows (Q4).
• Heavier cows (Q4) were associated with lower genetic merit for fertility traits.
• Fewer pregnancies per artificial insemination as heifers at first service were associated with ≥ 85% mature body weight cows (Q3, Q4).
• A greater proportion of Q1 cows had .1 bovine respiratory disease incidence as heifers.
• Insemination eligibility based on age and increased reproductive performance of heifers was negatively associated with primiparous milk production.

## Graphical Abstract

**Figure:**
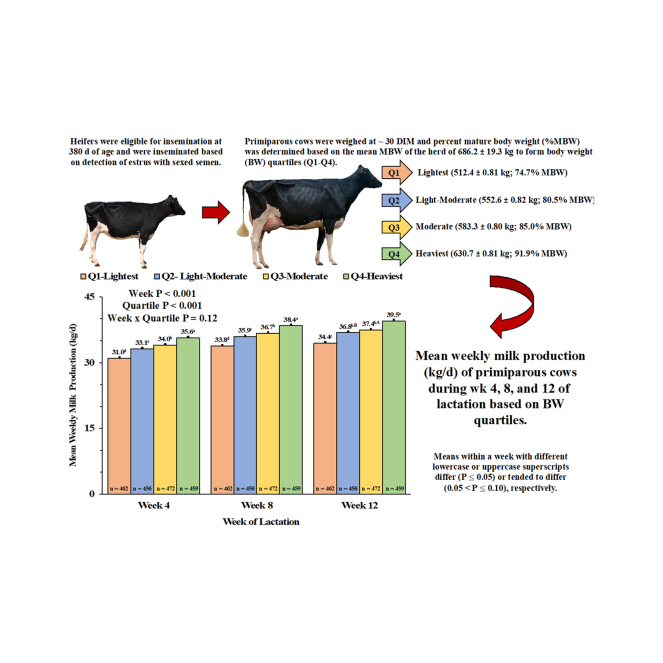
**Summary:** Body weight of primiparous cows is positively associated with milk production during first lactation. The association between insemination eligibility and reproductive performance of heifers with primiparous body weight at 30 days in milk and milk production, however, is not well characterized. We analyzed a data set of 1,849 primiparous Holstein cows that included respiratory disease and insemination records of heifers and subsequent body weight and milk production of primiparous cows. Insemination eligibility and reproductive performance, respiratory disease incidence, and genetic potential were associated with primiparous body weight at 30 days in milk and milk production at 4, 8, and 12 weeks of lactation.

From birth until calving, the average rearing cost of replacement heifers is approximately $2,500 with feed accounting for 50% of total rearing costs (Akins and Hagedorn, 2015; Karszes and Hill, 2020). Age at first calving (**AFC**) decreased by 2.4 mo in US Holsteins from 2006 to 2015 (Hutchison et al., 2017), which illustrates a trend in the dairy industry to achieve an earlier age at conception as a means to decrease rearing costs and thus generate income from milk production sooner. Although this strategy decreases rearing costs, it may not consider growth benchmarks for mature body size (**MBS**) of heifers to achieve their genetic potential for future milk production. Mature body size is defined as the mature body weight (**MBW**) and mature height (**MH**) of third and greater lactation cows in a herd measured at a consistent DIM. Current benchmarks for heifer growth are 55% MBW and 90% MH at first insemination, 94% MBW and 95% MH immediately precalving, and 85% MBW and 95% MH postcalving (Van Amburgh and Meyer, 2005; Van Amburgh et al., 1998; Heinrichs and Hargrove, 1987).

Despite these MBS benchmarks and measurement tools, only 36% of US dairy heifer growers record BW and ADG (NAHMS, 2011). Decreasing AFC from 25 to 21 mo by increasing reproductive efficiency decreased rearing costs by 18% under the assumption of adequate growth relative to MBS (Tozer and Heinrichs, 2001). With few US dairy farmers measuring heifer growth, however, adoption of an earlier age at conception was often done without regard to MBS benchmarks, thereby limiting heifers from achieving their genetic potential for milk production. Thus, the objective of this retrospective cohort study was to determine the association between insemination eligibility and reproductive performance of nulliparous heifers on BW at 30 DIM and milk production during wk 4, 8, and 12 of lactation of primiparous cows in a commercial dairy herd.

No human or animal subjects were used, so this analysis did not require approval by an Institutional Animal Care and Use Committee or Institutional Review Board. Data were extracted from a commercial dairy herd management program (DairyComp 305; Valley Ag Software) from a Holstein herd located in northwestern Iowa milking ~7,000 cows. Heifers were transported to a calf grower in Kansas at approximately 2 d of age for rearing until approximately 250 d of age, and heifers were then transported to a heifer grower in Nebraska. Nulliparous heifers were eligible for first insemination at 380 d of age and were inseminated based on detection of estrus with sexed semen. Pregnant nulliparous heifers were transported back to the dairy farm in Iowa approximately 110 d before calving. Average daily gain of heifers at the calf and heifer grower facilities was not available for analysis. The incidence of bovine respiratory disease (**BRD**) was recorded, and sick heifers were treated by trained personnel at the heifer grower facilities. Heifers were classified as either healthy (no BRD) or as having ≥1 BRD incidence before calving. Parent average PTA values for milk (kg), fat (kg), protein (kg), stature, feed saved (**FS**; kg), net merit dollars (**NM$**), productive life (**PL**), daughter pregnancy rate (**DPR**), and heifer conception rate (**HCR**) were estimated and extracted from the herd management software program.

The mean MBW of the herd was 686.2 ± 19.3 kg and was estimated by weighing a random subset of third and fourth lactation cows (n = 75) at 30 to 40 DIM. Primiparous cows were weighed at approximately 30 DIM, and the percent mature body weight (**%MBW**) was calculated for each cow as the BW at 30 DIM divided by the estimated mean MBW of the herd. Primiparous cows were milked thrice daily in an 80-stall rotary parlor. Milk weights were recorded at each milking to calculate the mean daily milk production (kg/d). The mean weekly milk production (kg/d) of primiparous cows at wk 4, 8, and 12 of lactation was extracted from the herd management software program and was calculated as the mean of the mean daily milk production during each week.

Initially, records from 2,962 primiparous Holstein cows that calved between May 2018 and January 2021 with BW at 30 DIM were extracted and analyzed. Primiparous cows were removed from the data set if they were labeled as do not breed (n = 184), had a gestation length of <250 or >300 d as a nulliparous heifer (n = 33), or did not have a mean weekly milk production record at wk 4, 8, or 12 of lactation (n = 462). Outlier and influential data points (n = 168) and heifers inseminated with conventional semen (n = 242) or to timed AI after a CIDR-Synch protocol (n = 24) were excluded from the data set because of the potential differences in pregnancies per AI (**P/AI**) due to semen type and insemination method (Chebel and Cunha, 2020). The final data set used for the analysis included 1,849 primiparous Holstein cows. Quartiles based on BW at 30 DIM and %MBW of primiparous cows were created in ascending order as follows: **Q1** (lightest; n = 462), **Q2** (light-moderate; n = 456), **Q3** (moderate; n = 472), and **Q4** (heaviest; n = 459) using the PROC RANK procedure of SAS (Table 1).

**Table 1:** Mean (±SEM) BW (kg) at 30 DIM, mature body weight (MBW, %), age at first calving (AFC, d), and parent average PTA values of primiparous Holstein cows based on weight quartiles (Q1–Q4) at 30 DIM

Item	BW quartile			
	Q1 n = 462	Q2 n = 456	Q3 n = 472	Q4 n = 459
BW at 30 DIM (kg)	512.4^d^ ± 0.81	552.6^c^ ± 0.82	583.3^b^ ± 0.80	630.7^a^ ± 0.81
MBW^1^ (%)	74.7^d^ ± 0.001	80.5^c^ ± 0.001	85.0^b^ ± 0.001	91.9^a^ ± 0.001
AFC (d)	674.6^d^ ± 1.25	681.8^c^ ± 1.25	688.2^b^ ± 1.24	694.6^a^ ± 1.25
PTA^2^				
Milk (kg)	173.1^b^ ± 9.75	188.6^ab^ ± 9.83	179.2^b^ ± 9.67	215.0^a^ ± 9.79
Fat (kg)	12.8^b^ ± 0.27	13.3^b^ ± 0.27	13.1^b^ ± 0.26	14.4^a^ ± 0.27
Protein (kg)	7.7^b^ ± 0.24	7.9^b^ ± 0.24	7.9^b^ ± 0.24	9.1^a^ ± 0.24
Stature	−0.56^c^ ± 0.03	−0.52^bc^ ± 0.03	−0.46^b^ ± 0.03	−0.29^a^ ± 0.03
Feed saved (kg)	31.9^a^ ± 2.0	24.6^b^ ± 2.0	13.4^c^ ± 2.0	5.7^d^ ± 2.0
Net merit $ (NM$)	274.7^A^ ± 3.2	272.7^AB^ ± 3.2	263.4^B^ ± 3.1	270.4^AB^ ± 3.2
Productive life (PL)	2.4^a^ ± 0.04	2.2a–d, A,B ± 0.04	2.1a–d, A,B ± 0.04	1.9^d^ ± 0.04
Daughter pregnancy rate (DPR)	0.37^a^ ± 0.05	0.27a–d, A,B ± 0.05	0.26^ab^ ± 0.05	0.11a–d, A,B ± 0.05
Heifer conception rate (HCR)	0.03^a^ ± 0.04	0.0^a^ ± 0.04	−0.08^ab^ ± 0.04	−0.16^b^ ± 0.04

a–d Within a row, means with different lowercase superscripts differed (*P* ≤ 0.05).

A,B Within a row, means with different uppercase superscripts tended to differ (0.05 < *P* ≤ 0.10).

1 Percent mature body weight (%MBW) was calculated as the recorded weight of primiparous cows at 30 DIM divided by the MBW of the herd of 686.2 kg determined by the mean weight of a random sample of third and fourth lactation cows (n = 75) at 30 to 40 DIM.

2 Parent average PTA estimated from DairyComp 305 software (Valley Ag Software).

All statistical analyses were performed using SAS computational software version 9.4 for Microsoft Windows (SAS Institute Inc.). Outlier and influential data points were identified using external studentized residuals and Cook's distance using the PROC REG procedure of SAS with the OUTPUT statement and keywords RSTUDENT and COOKD, respectively. Data points were removed when they were more than 3 standard deviations from the predicted value, when Cook's distance was >0.0002, or both. Differences in descriptive data among quartiles including weight at 30 DIM, %MBW, AFC, and PTA values were analyzed using the PROC GLM procedure of SAS with a model that included the fixed effect of quartile, and data are presented as the least squares means ± standard error of the mean (**SEM**) using the LSMEANS statement. Binary response data (i.e., P/AI at first insemination and proportion of cows with ≥1 BRD incidence as heifers) were analyzed by logistic regression using the GLIMMIX procedure of SAS, and data are presented as least squares means ± SEM using the LSMEANS statement. The model for P/AI at first insemination included the fixed effects of quartile, BRD incidence, and the quartile × BRD incidence interaction, and the model for BRD incidence as heifers included the fixed effect of quartile.

The association between milk production at wk 4, 8, and 12 of lactation as primiparous cows based on BW quartiles and ≥1 BRD incidence as a nulliparous heifer was analyzed using a repeated measures ANOVA using the repeated statement in the GLM procedure of SAS. The model included the within-subject effects and interactions of week, week × quartile, week × BRD incidence, and week × quartile × BRD incidence. The between-subject effects included quartile, BRD incidence, and the quartile × BRD incidence interaction. A multivariate ANOVA (MANOVA) rather than a univariate approach for within-subject effects and interactions was used because of the lack of sphericity determined using the Mauchly's test of sphericity. Milk production (kg) at wk 4, 8, and 12 are presented as least squares means ± SEM using the LSMEANS statement in SAS. A significant difference in the fixed effects was declared when *P* ≤ 0.05 and a statistical tendency was declared when 0.05 < *P* ≤ 0.10.

Descriptive data of quartiles including BW at 30 DIM, %MBW, AFC, and PTA values are presented in Table 1. By design, mean BW at 30 DIM and %MBW increased (*P* < 0.001) linearly by quartile. Only Q3 and Q4 cows achieved the industry benchmark of ≥85% MBW postcalving (Van Amburgh and Meyer, 2005). Overall, AFC increased (*P* < 0.001) with increasing quartile with Q4 cows calving 20.0 ± 1.77 d later than Q1 cows. Overall, Q4 cows had greater (*P* = 0.01) genetic potential for milk production than Q1 and Q3 cows, but not Q2 cows.

The PTA values for DPR and HCR differed (*P* < 0.001) among quartiles, with Q1 cows having greater genetic potential for DPR and HCR than Q4 cows. Parent average (Kuhn et al., 2006) and genomic merit (Veronese et al., 2019) for DPR were positively associated with P/AI at first insemination in nulliparous heifers. Veronese et al. (2019) reported negative correlations between genomic DPR and HCR with genomic milk yield in Holstein heifers.

The PTA value for stature differed (*P* < 0.001) among quartiles with Q4 cows having greater genetic potential for height than Q1, Q2, and Q3 cows. Across dairy breeds, % MBW recommendations are constant, but variability exists within breeds based on genetic variation in body size (Koenen and Groen, 1998; Hoffman, 2007). Hurst et al. (2022) reported a 21.6 kg difference in predicted BW at 400 d of age between Holstein heifers in the lowest and highest quartiles based on BW composite index. Thus, it is important to consider the genetic potential for body size of individual heifers when evaluating heifer growth and subsequent milk production because heifers are often genetically selected to be smaller and more feed efficient.

Overall, 27.4% of nulliparous heifers had ≥1 BRD incidence before first calving. There was an association (*P* = 0.006) between BW quartile and the proportion of heifers with ≥1 BRD incidence in which Q1 cows had a 9 to 11 percentage point greater incidence of BRD as heifers than Q2, Q3, and Q4 cows (Figure 1A). Primiparous cows with no BRD incidence when they were heifers weighed more (*P* < 0.001) at 30 DIM than cows with ≥1 BRD incidence when they were heifers (572.1 ± 1.27 vs. 563.7 ± 2.06 kg). Based on a meta-analysis, heifers with a BRD incidence during calfhood had decreased ADG of 0.067 kg/d (Buczinski et al., 2021), and heifers treated for BRD postweaning weighed 14.3 kg less and were 1.7 cm shorter at the withers than healthy heifers at 13 mo of age (Stanton et al., 2012). The number of times a heifer was treated for BRD (1, 2, or 3+) was not associated with predicted BW, but healthy heifers were associated with a greater predicted BW at 400 d than heifers with ≥1 BRD incidence (Hurst et al., 2021).

**Figure 1:**
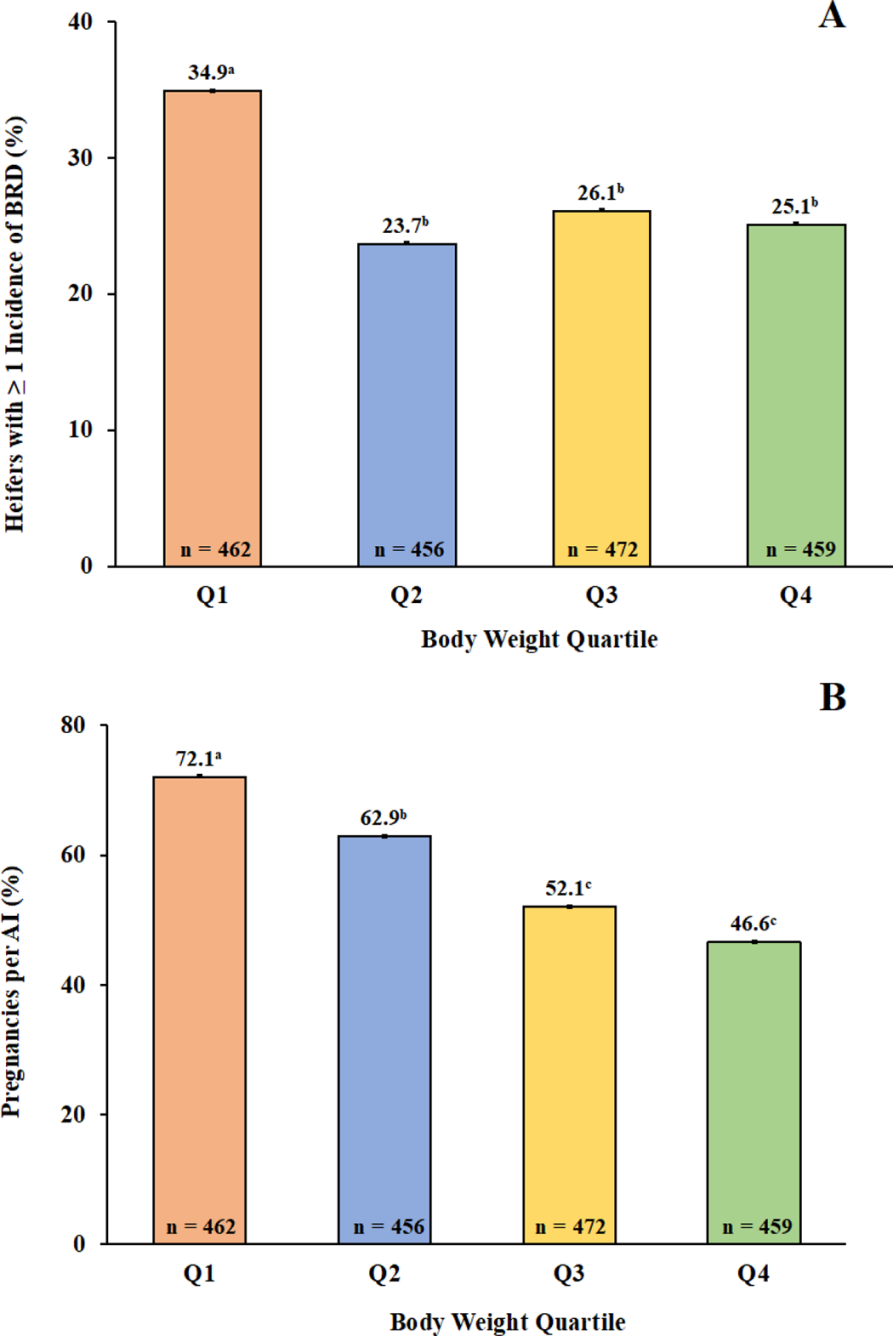
(A) Incidence of bovine respiratory disease (BRD) as nulliparous heifers by BW quartile (Q1–Q4) at 30 DIM as primiparous cows; and (B) pregnancies per AI (P/AI; %) at first insemination as heifers after detection of estrus and AI with sexed semen by BW quartile (Q1–Q4) at 30 DIM as primiparous cows. Percentages with different lowercase letters differed (*P* ≤ 0.05).

Overall, P/AI of nulliparous heifers at first insemination based on a detected estrus and using sexed semen was 58.4%, and there was no interaction (*P* = 0.42) between quartile and BRD incidence on P/AI at first insemination as heifers. Further, there was no association (*P* = 0.35) between BRD incidence and P/AI at first insemination as nulliparous heifers (58.2% for healthy heifers vs. 60.1% for heifers with ≥1 BRD incidence). Primiparous cows with ≥1 BRD incidence as a heifer had a decreased (*P* = 0.02) AFC than primiparous cows that were healthy as heifers (685.7 ± 0.76 vs. 682.3 ± 1.23 d). The decreased AFC of heifers with ≥1 BRD incidence in our study likely occurred because insemination eligibility was primarily based on age rather than MBS and because Q1 cows had the greatest proportion of cows with ≥1 BRD incidence (Figure 1A) and P/AI at first insemination as heifers (Figure 1B).

Body weight quartile was associated (*P* < 0.001) with P/AI at first service as nulliparous heifers with Q1 cows having approximately 9 to 26 percentage points more P/AI at first insemination as heifers than Q2, Q3, and Q4 cows (Figure 1B). By conceiving and calving approximately 20 d earlier than Q4 cows, Q1 cows had fewer days on feed and thus a shorter growth period as heifers before beginning first lactation to achieve ≥85% MBW postcalving. By contrast, over half of Q4 cows failed to conceive at first insemination when they were heifers, thereby allowing for one or more estrous cycles to grow before conceiving at a later insemination. Because heifers were eligible for first insemination primarily based on age, the separation of heifers into weight quartiles in this analysis was largely due to differences in genetic merit for fertility traits among heifers.

For within-subject effects, there was a tendency for an interaction among week × quartile × BRD incidence (*P* = 0.09) but there was no interaction between week × quartile (*P* = 0.12) and week × BRD incidence (*P* = 0.91) associated with primiparous milk production at wk 4, 8, and 12 of lactation. Milk production increased (*P* < 0.001) from wk 4 (33.4 ± 0.11 kg), 8 (36.1 ± 0.11 kg), and 12 (36.9 ± 0.12 kg) of lactation. For between-subject effects, there was no interaction of quartile × BRD incidence (*P* = 0.44) associated with milk production at wk 4, 8, and 12 of lactation. Further, there was no association of BRD incidence (*P* = 0.12) with milk production at wk 4 (33.2 ± 0.12 kg vs. 33.6 ± 0.20 kg), 8 (36.0 ± 0.12 kg vs. 36.4 ± 0.20 kg), and 12 (36.9 ± 0.13 kg vs. 37.1 ± 0.21 kg) of lactation for primiparous cows that were healthy or ≥1 incidence of BRD as heifers, respectively. Incidence of BRD likely affects primiparous milk production indirectly through decreased growth during the rearing period and could be underestimated because of survivorship bias (Stanton et al., 2012; Adams and Buczinski, 2016; Hurst et al., 2022).

There was a positive association (*P* < 0.001) between BW quartile and primiparous milk production at wk 4, 8, and 12 of lactation (Figure 2) with Q4 cows yielding approximately 5 kg per cow/d more milk than Q1 cows (Figure 2). Based on a shortened growth period to achieve 85% MBW, Q1 cows were unable to achieve their genetic potential for milk production because energy was likely partitioned to growth rather than to lactation. Thus, insemination eligibility and reproductive performance, calfhood BRD incidences, and genetic potential for milk production, body size, and fertility traits were all associated with primiparous milk production in the present analysis.

**Figure 2:**
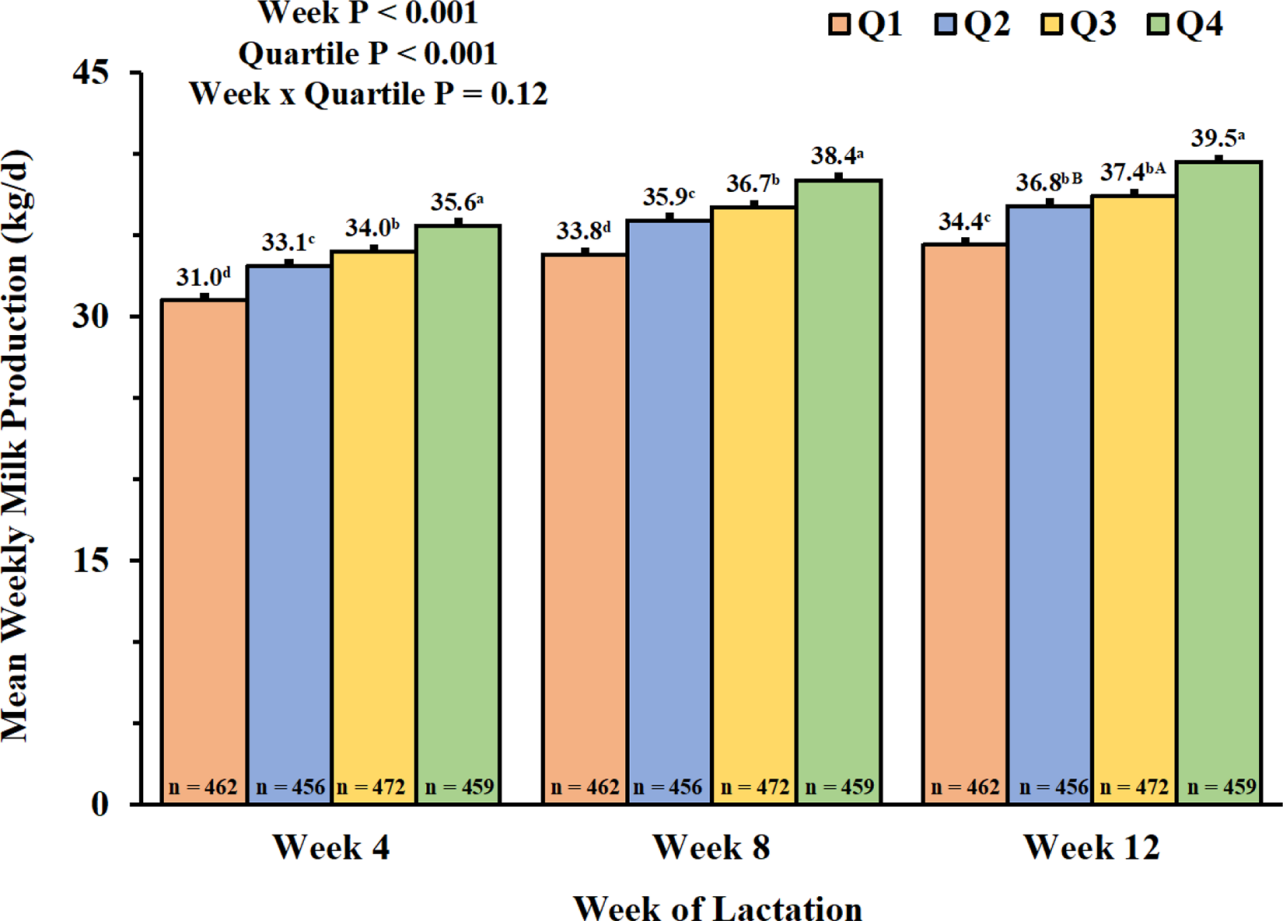
Mean (±SEM) weekly milk production (kg/d) at wk 4, 8, and 12 of lactation of primiparous Holstein cows by BW quartile at 30 DIM (Q1–Q4). Means with different lowercase letters differed (*P* ≤ 0.05). Means with different uppercase letters tended to differ (0.05 < *P* ≤ 0.10).

In early observational studies, greater weight at first calving was associated with increased milk production during first lactation and explained more of the variance in milk production than AFC (Fisher et al., 1983; Keown and Everett, 1986; Clark and Touchberry, 1962). In a study from New Zealand (Handcock et al., 2019), heavier BW heifers had increased energy-corrected milk production as primiparous as well as accumulated third-parity milk production. Han et al. (2021) investigated the association of BW at first calving with first lactation 305-d milk yield through production records of heifers reared within 2 university herds. Heifers in the top 60% of BW at first calving had greater first lactation 305-d milk yield, but there was no difference in first lactation 305-d milk yield among heifers within the top 60% BW at first calving (Han et al., 2021). Further, Han et al. (2021) reported that heavier heifers had increased milk production, but only during first lactation, and they also had an increased risk of culling. By contrast, Handcock et al. (2020) reported that heifers in New Zealand predicted for heavier BW were associated with increased survivability than lighter BW heifers.

This data set provides insights into the relationship between insemination eligibility and reproductive performance of nulliparous heifers with subsequent BW at 30 DIM and primiparous milk production but has several limitations. Genomic predictions are more reliable than parent average PTA values to account for the genetic variance of heifers but this herd was unfortunately not genomically testing their heifers. Further, genetic potential for body size varies among heifers, and this variation is difficult to account for using a mean %MBW of a herd. With few US dairy herds measuring the body size of heifers, however, the use of a mean %MBW for a herd serves as a basis.

In conclusion, insemination eligibility (380 d of age) and reproductive performance of nulliparous heifers were associated with BW at 30 DIM and subsequent milk production at wk 4, 8, and 12 of lactation in primiparous cows. The lightest primiparous cows were associated with the greatest proportion of cows ≥1 BRD incidence as heifers. Further, the lightest primiparous cows on average were associated with greater genetic potential for DPR and HCR than the heaviest primiparous cows. Phenotypically, the lightest primiparous cows were associated with approximately 26 percentage points more P/AI at first service as nulliparous heifers than the heaviest primiparous cows. With the most P/AI at first service as nulliparous heifers, the lightest primiparous cows had a shorter growth period with fewer days on feed as heifers. At wk 4, 8, and 12 of lactation, the lightest primiparous cows were associated with approximately 5 kg per cow/d less milk compared with the heaviest primiparous cows. Randomized-controlled studies are needed to further understand the effects of insemination eligibility and reproductive performance, calfhood disease incidence, and genetic potential on heifer growth, survivability, and subsequent milk production.

## References

[bib1] Adams E.A., Buczinski S. (2016). Short communication: Ultrasonographic assessment of lung consolidation postweaning and survival to the first lactation in dairy heifers. J. Dairy Sci..

[bib2] Akins M.S., Hagedorn M.A. (2015). https://fyi.extension.wisc.edu/dairy/files/2015/10/2015-Cost-of-Raising-Replacements-Factsheet-Final.pdf.

[bib3] Buczinski S., Achard D., Timsit E. (2021). Effects of calfhood respiratory disease on health and performance of dairy cattle: A systematic review and meta-analysis. J. Dairy Sci..

[bib4] Chebel R.C., Cunha T. (2020). Optimization of timing of insemination of dairy heifers inseminated with sex-sorted semen. J. Dairy Sci..

[bib5] Clark R.D., Touchberry R.W. (1962). Effect of body weight and age at calving on milk production in Holstein cattle. J. Dairy Sci..

[bib6] Fisher L.J., Hall J.W., Jones S.E. (1983). Weight and age at calving and weight change related to 1st lactation milk-yield. J. Dairy Sci..

[bib7] Han L., Heinrichs A.J., De Vries A., Dechow C.D. (2021). Relationship of body weight at first calving with milk yield and herd life. J. Dairy Sci..

[bib8] Handcock R.C., Lopez-Villalobos N., McNaughton L.R., Back P.J., Edwards G.R., Hickson R.E. (2019). Positive relationships between body weight of dairy heifers and their first-lactation and accumulated three-parity lactation production. J. Dairy Sci..

[bib9] Handcock R.C., Lopez-Villalobos N., McNaughton L.R., Back P.J., Edwards G.R., Hickson R.E. (2020). Body weight of dairy heifers is positively associated with reproduction and stayability. J. Dairy Sci..

[bib10] Heinrichs A.J., Hargrove G.L. (1987). Standards of weight and height for Holstein heifers. J. Dairy Sci..

[bib11] Hoffman P.C. (2007). Proc. Western Dairy Management Conf. Reno, NV.

[bib12] Hurst T.S., Lopez-Villalobos N., Boerman J.P. (2021). Predictive equations for early-life indicators of future body weight in Holstein dairy heifers. J. Dairy Sci..

[bib13] Hurst T.S., Neves R.C., Boerman J.P. (2022). Early life indicators of first lactation milk yield and the effect of treatment for bovine respiratory disease on survivability and risk of pregnancy in Holstein dairy cattle. Vet. J..

[bib14] Hutchison J.L., VanRaden P.M., Null D.J., Cole J.B., Bickhart D.M. (2017). Genomic evaluation of age at first calving. J. Dairy Sci..

[bib15] Karszes J., Hill L. (2020). https://dyson.cornell.edu/wp-content/uploads/sites/5/2020/09/Dairy-Replacement-Costs-Writeup-Final1-VD.pdf.

[bib16] Keown J.F., Everett R.W. (1986). Effect of days carried calf, days dry, and weight of 1st calf heifers on yield. J. Dairy Sci..

[bib17] Koenen E.P.C., Groen A.F. (1998). Genetic evaluation of body weight of lactating Holstein heifers using body measurements and conformation traits. J. Dairy Sci..

[bib18] Kuhn M.T., Hutchison J.L., Wiggans G.R. (2006). Characterization of Holstein heifer fertility in the United States. J. Dairy Sci..

[bib19] NAHMS (2011).

[bib20] Stanton A.L., Kelton D.F., LeBlanc S.J., Wormuth J., Leslie K.E. (2012). The effect of respiratory disease and a preventative antibiotic treatment on growth, survival, age at first calving, and milk production of dairy heifers. J. Dairy Sci..

[bib21] Tozer P.R., Heinrichs A.J. (2001). What affects the costs of raising replacement dairy heifers: A multiple-component analysis. J. Dairy Sci..

[bib22] Van Amburgh M., Meyer M. (2005). Dairy Calves and Heifers: Integrating Biology and Management.

[bib23] Van Amburgh M.E., Galton D.M., Bauman D.E., Everett R.W., Fox D.G., Chase L.E., Erb H.N. (1998). Effects of three prepubertal body growth rates on performance of Holstein heifers during first lactation. J. Dairy Sci..

[bib24] Veronese A., Marques O., Moreira R., Belli A.L., Bisinotto R.S., Bilby T.R., Penagaricano F., Chebel R.C. (2019). Genomic merit for reproductive traits. I: Estrous characteristics and fertility in Holstein heifers. J. Dairy Sci..

